# Interaction of allergic airway inflammation and innate immunity: hygiene and beyond

**DOI:** 10.1186/1745-6673-3-S1-S3

**Published:** 2008-02-27

**Authors:** Christoph Beisswenger, Robert Bals

**Affiliations:** 1 Department of Microbiology, University of Pennsylvania School of Medicine, Philadelphia, PA 19104, USA; 2 Department of Internal Medicine, Division for Pulmonary Diseases, Philipps-Universtät Marburg, 35043 Marburg, Germany

## Abstract

The lung is constantly exposed to the environment and its microbial components. Infections of the respiratory tract are amongst the most common diseases. Several concepts describe how this microbial exposure interacts with allergic airway disease as it is found in patients with asthma. Infections are classical triggers of asthma exacerbations. In contrast, the hygiene hypothesis offers an explanation for the increase in allergic diseases by establishing a connection between microbial exposure during childhood and the risk of developing asthma. This premise states that the microbial environment interacts with the innate immune system and that this interrelation is needed for the fine-tuning of the overall immune response. Based on the observed protective effect of farming environments against asthma, animal models have been developed to determine the effect of specific bacterial stimuli on the development of allergic inflammation. A variety of studies have shown a protective effect of bacterial products in allergen-induced lung inflammation. Conversely, recent studies have also shown that allergic inflammation inhibits antimicrobial host defense and renders animals more susceptible to bacterial infections. This paper focuses on examples of animal models of allergic disease that deal with the complex interactions of the innate and adaptive immune system and microbial stressors.

## 

The lung is constantly exposed to the environment and its microbial components. Infections of the respiratory tract are amongst the most common diseases. Several concepts describe how this microbial exposure interacts with allergic airway disease as it is found in patients with asthma. Infections are classical triggers of asthma exacerbations. Infections of the respiratory tract, especially with various viruses, are common causes of exacerbation of asthma [1,2].

The hygiene hypothesis offers an explanation for rising rates of allergic diseases such as asthma in modern westernized societies. The core of this hypothesis is the complex interaction of the microbial environment and the innate immune system in the childhood of individuals. In modern societies, different factors such as small family size, high antibiotics use, and good sanitation contribute to higher living standards and life expectancy [3]. As a result, regulatory mechanisms that are activated by the interaction with the microbial environment and that are needed to balance the adaptive immune response might be disturbed. Hygienic environments typical for modern societies might lack the stimuli that are needed to adjust the Th1 and Th2 cell mediated adaptive immune responses finally resulting in an increased prevalence for both Th1-mediated and Th2-mediated diseases [4].

The observed protective effect of farming environments in connection with environmental exposure to endotoxin may have a crucial role in the development of tolerance to ubiquitous allergens found in natural environments against asthma [5]. This observation led to the development of animal models to determine the effect of specific bacterial stimuli on the development of allergic inflammation. For instance, several studies have shown a protective effect of TLR9 agonist on allergic inflammation of the respiratory tract. Activation of TLR9 promoted the development of Th1 cell response in vitro and in vivo. Treatment with TLR9 agonists inhibited the development of airway hyperresponsiveness, mucus production, and airway eosinophil infiltration in mouse asthma models [6,7]. Furthermore, another study using a murine model of asthma showed that interperitonal administration of a synthetic TLR2 ligand ameliorates established allergic airway inflammation [8]. The authors claimed that the key therapeutic approach is to achieve a balance between Th2 and Th1 cytokines by decreasing Th2 cytokines without a large increase in Th1 response. In addition to TLR2 agonists, animal studies have also demonstrated that LPS signalling suppresses allergic inflammation of the respiratory tract. Effects of LPS were mediated by TLR4 [9] and were independent of IL-12 production by dendritic cells [10].

In the last years, it has become clear that the appropriate bacterial composition of the human microflora is a factor in protection from allergy and asthma and is needed for an adequate Th1 / Th2 balance [11]. Interestingly, in a mouse model of asthma, oral treatment with probiotic organisms inhibited the allergic response in a TLR9 dependent manner [12]. Oral treatment with live *Lactobacillus reuteri* attenuated the influx of eosinophils into the respiratory tract and was accompanied by levels of Th2 cytokines.

The studies outlined above deal with the impact of bacterial stressors that preferentially promote a Th1 cell mediated response on the development of Th2 mediated allergic disease such as asthma. Conversely, a largely unknown field is the influence of established allergic diseases on infectious diseases that require an appropriate innate or Th1 cell mediated immune response.

A recent study investigated the effect of an established allergic inflammation of the lung on the innate host defense during bacterial infection of the respiratory tract [13]. The main finding was that the adaptive immune system modulates the functions of the pulmonary innate immune system and that allergic inflammation of the lung inhibits pulmonary antimicrobial host defense. Taking advantage of an animal model of allergic airway disease, it was shown that Th2-type inflammation in the lung results in a suppressed antibacterial host defense after infection with *Pseudomonas aeruginosa*. Mice with allergic airway inflammation had more viable bacteria and reduced levels of pro-inflammatory cytokines and antimicrobial peptides in their lung. Furthermore, the influx of neutrophils into the respiratory tract was significantly diminished in asthmatic animals.

The suppressive effect of the Th2 milieu was also shown in an in vitro model using differentiated human airway epithelia tissue [13]. The Th2 cytokines IL-4 and IL-13 showed an inhibitory effect on the antimicrobial activity of the airway epithelium, as airway epithelial cells were unable to kill bacteria when incubated with these cytokines. This inhibitory effect was accompanied by a reduction in expression of the antimicrobial peptide human β-defensin 2. These results are consistent with studies showing that the cytokine milieu of atopic dermatitis prevents induction of innate immune response factors such as antimicrobial peptides, IL-8 and iNos. Additionally, a higher susceptibility of patients with atopic dermatitis to skin infections was observed [14,15]. However, the exact mechanism how the Th2 milieu inhibits the antimicrobial host defense is not clear yet. Interestingly, it has been demonstrated that Th2 cytokines inhibit the TNF-α/NF-κB system through activation of STAT6 which acts as a transcriptional inhibitor of NF-κB-dependent gene expression [16]. Given that many genes induced during the innate immune response are under control of NF-κB, inhibition of NF-κB signalling by Th2 cytokines could explain the suppressed innate immune response to bacterial infection in presence of an allergic inflammation. A complete understanding of how Th2 cytokines suppress antimicrobial activity of the epithelial cells remains to be determined.

These results provide evidence that the adaptive immune system closely interacts with the innate immune system and that the adaptive immune response influences the innate host defense (Figure 1). The Th2-dependent suppression of the pulmonary innate immune system could result in increased susceptibility to colonization and infection. Chronic colonization could be a risk factor for asthmatic lung disease. For instance, patients with asthma are often chronically colonized with *Mycoplasma* and *Chlamydia* species [17].

**Figure 1:**
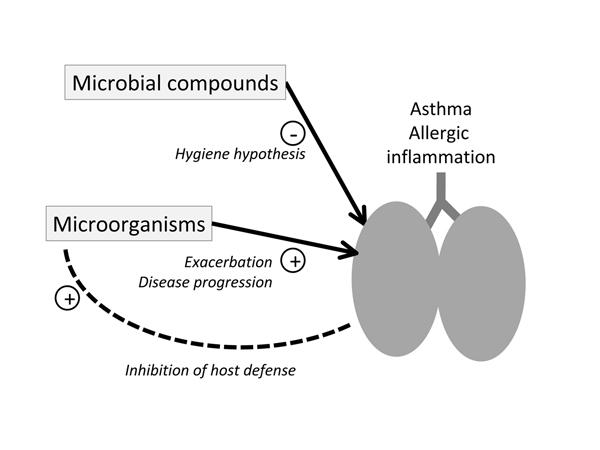
Exposure of the lung with microorganisms modulates allergic airway inflammation in several ways. Inversely, allergic airway inflammation impacts on host defense. The association of decreased host defense in asthma with increased susceptibility to colonization with specific microorganisms might further contribute to disease development.

## Conclusion

The development of allergic diseases is a complex process. In recent years, it became clear that the interaction of the microbial environment with the innate and adaptive immune system during childhood is crucial for a well balanced immune system. The combination of animal models of allergic disease with infection models is a useful tool to further study the interactions between these processes and is a starting point for new therapeutic strategies.

## Competing interests

The authors declare that they have no competing interests.
